# Infective Vaginal Discharge among Women of the Reproductive Age in the Outpatient Department of a Primary Care Centre

**DOI:** 10.31729/jnma.8432

**Published:** 2024-02-29

**Authors:** Shailaja Khadka, Ratna Khatri, Raina Chaudhary

**Affiliations:** 1 Department of Obstetric and Gynaecology, Nepalese Army Institute of Health Sciences, Sanobharyang, Kathmandu, Nepal; 2 Department of Microbiology, Nepalese Army Institute of Health Sciences, Sanobharyang, Kathmandu, Nepal

**Keywords:** *bacterial*, *candidiasis*, *prevalence*, *vaginitis*

## Abstract

**Introduction:**

Infective vaginal discharge is the most common complaint of the women of reproductive age group attending gynaecology outpatient department. Vaginal discharge may be normal or abnormal. Infective vaginal discharge is usually related to one of the three conditions, like bacterial vaginosis, vulvovaginal candidiasis, and trichomoniasis. This study aimed to find out the prevalence of infective vaginal discharge among women of the reproductive age in the outpatient department of a primary care centre.

**Methods:**

A descriptive cross-sectional study was carried out among women of the reproductive age group visiting the outpatient Department of the primary care hospital from 1 March 2022 to 1 August 2022 after obtaining ethical approval from the Institutional Review Committee. A convenience sampling method was used. The point estimate was calculated at a 95% Confidence Interval.

**Results:**

Among 138 patients, infective vaginal discharge was found in 42 (30.43%) (21.32-39.54, 95% Confidence Interval). Bacterial vaginosis was seen in 22 (52.38%), *Candida* was seen in 13 (30.95%), and *Trichomonas* was seen in 7 (16.66%) cases.

**Conclusions:**

The prevalence of infective vaginal discharge was lower as compared to other studies done in similar settings.

## INTRODUCTION

Infective vaginal discharge is the most common complaint of the women of reproductive age group attending gynaecology outpatient department (OPD). Approximately 10 million outpatient department visits each year attributes to vaginal discharge.^1^ Vaginal discharge may be normal or abnormal.^2^ Infective vaginal discharge may be green, yellow, brown or red coloured with foul smell, pruritic, irritation, dysuria or dyspareunia.^3^ Pathological symptoms include infectious causes, such as bacterial vaginosis, vulvovaginal candidiasis and trichomoniasis, corresponding to 90% of cases.^4^ Symptomatic vaginal discharge is caused by inflammation due to infection of the vaginal mucosa.^5^

The physiological discharges are clear or whitish and variable throughout the menstrual cycle and composed of microbiota and cervical fluids. It is one of the frequent gynaecological complaints confused by women with infectious symptoms, especially those caused by fungi.^4^ The conditions can be misdiagnosed or maltreated.

This study aimed to find out the prevalence of infective vaginal discharge among women of the reproductive age in the outpatient department of a primary care centre.

## METHODS

A descriptive cross-sectional study was conducted among women of the reproductive age group in the outpatient Department of Obstetrics and Gynaecology of Military Hospital Itahari, after obtaining ethical approval from the Institutional Review Committee (Reference number: 245). Data from 1 March 2022 to 1 August 2022 was collected after taking written informed consent from the individual patients. reproductive-age women of 15-49 years giving consent were included in the study. Women who did not understand the questions properly, those who had used antibiotics or vaginal medication, bleeding per vaginum were excluded. A convenience sampling method was used. The sample size was calculated by using the following formula:


$$\begin{array}{ll} \text{n} & ={\text{Z}}^{2}\times \frac{\text{p}\times \text{q}}{{\text{e}}^{\text{2}}}\\ & =1.96^{2}\times \frac{0.068\times 0.932}{0.05^{2}}\\ & =98 \end{array}$$

Where,

n = minimum required sample size
Z = 1.96 at 95% Confidence Interval (CI)
p = prevalence from the previous study, 6.8%^6^
q = i-p
e = margin of error, 5%

The calculated sample size was 98. However, the final sample size taken was 138.

A detailed clinical history and a thorough examination of all the women were done. A sterilized Cusco's bivalve self-retaining vaginal speculum was inserted into the vagina to visualise the vagina and cervix. The amount, colour, character and smell of the vaginal discharge were noted. Three sterile swabs were then collected. The swabs of vaginal discharge were sent in (i) normal saline (ii) a sterile vial, and (iii) a third swab was used for making smears. For *Candida:* KOH preparation: A drop of 10% KOH was added to the vaginal secretions taken on a clean glass slide and mounted with a cover slip. *Candida* was identified as highly retractile, round or oval budding yeast cells also Gram stained vaginal smears were examined which showed Gram-positive budding yeast cells with pseudohyphae.^6^ For *Trichomonas:* specimens for the wet smear examination were taken from the posterior fornix with a sterilized cotton swab and mixed with a drop of normal saline on a clean glass slide. A cover slip was mounted on the glass slide and the wet film was examined immediately under a microscope for flagellate organisms with characteristic motility.^7^ For bacterial vaginosis, detection diagnosis was made if three out of four Amsel's criteria.^8^

Data were entered and analyzed using IBM SPSS Statistics version 22.0. The point estimate was calculated at a 95% CI.

## RESULTS

Among 138 patients, the prevalence of infective vaginal discharge was 42 (30.43%) (21.32-39.54, 95% CI). Bacterial vaginosis was seen in 22 (52.38%) (Table 1).

**Table 1:** Distribution of infective vaginal discharge among women of reproductive age group (n = 42).

Infections	n <%)
Bacterial vaginosis	22 (52.38)
*Candida*	13(30.95)
*Trichomonas*	7(16.66)

Vaginal discharge was the most common presenting complaint in maximum cases 25 (59.52%) followed by vaginal itching and discharge 13 (30.95%).

**Table 2:** Presenting complaints of the women with infective vaginal discharge (n = 42).

Presenting complaints	n (%)
Vaginal discharge	25 (59.52)
Vaginal discharge & itching	13 (30.95)
Vaginal itching	2 (4.76)
Foul smelly discharge	2 (4.76)

Infections were found more in multiparous women bacterial vaginosis 19 (45.24%), *Candida* 11 (26.19%), and *Trichomonas* 6 (14.28%) (Table 3).

**Table 3:** Distribution of pathogens according to parity (n = 42).

	Bacterial vaginosis n (%)	Candida n (%)	Trichomonas n (%)
Multiparous	19 (45.23)	11 (26.19)	6 (14.28)
Primiparous	3 (7.14)	1 (2.38)	1 (2.38)
Nulliparous	-	1 (2.38)	-

## DISCUSSION

Among 138 patients, the prevalence of infective vaginal discharge was 42 (30.43%) which was found to be lower than the studies conducted in similar other settings. In a similar study conducted in India prevalence was found to be 48.6%.^7^ Similarly, in two past studies, the prevalence of infective vaginal discharge was 55.6% and 63.8% respectively.^8,9^ However, the prevalence in a study conducted in China was found to be 13.4%.^10^

In this study, most of the presenting complaints of the patients were vaginal discharge which was 25 (59.52%), followed by vaginal itching with discharge 13 (30.95%) and foul smelly discharge 2 (4.76%). Similar findings of vaginal discharge of 25% were found in a previous study.^10^ In this study prevalence of itchy vaginal discharge was 13 (30.9%) which is similar to a study conducted in Nigeria with a prevalence of 41.3%.^11^ In this study maximum, the number of organisms found in multiparous women were bacterial vaginosis 19 (45.24%), trichomoniasis 6 (14.29%) and candida 11 (26.19%) whereas only 1 (2.38%) case of candida was seen in nulliparous women. The previous study conducted showed that the prevalence of Candida and bacterial vaginosis was higher with multiparity 463 (92.6%).^7^

There are a few limitations in our study. This study includes women from the Nepal army or dependent on the Nepal army belonging to the single primary care centre and the sample size is also small. So, the result of our study cannot be generalized to the whole community.

## CONCLUSIONS

The prevalence of infective vaginal discharge was lower as compared to other studies done in similar settings. As most of the cases presenting with vaginal discharges were not positive for any pathogens, so while treating the patient it is important to look at the history, signs, and symptoms and if possible establish the microbiological diagnosis for the vaginal discharge and then provide the treatment. Antibiotic therapy should be given cautiously to cover bacterial and candidal infections. Routine microbiological diagnosis for the cause of vaginal discharge is recommended wherever facilities are available.
